# Contemporary practice and resource availability for genetic testing in paediatric hypertrophic cardiomyopathy

**DOI:** 10.1136/jmg-2025-110696

**Published:** 2025-05-16

**Authors:** Christoph Sandmann, Sabine Klaassen, Juan Pablo Kaski, Gabrielle Norrish, On behalf of the International Paediatric Hypertrophic Cardiomyopathy Consortium investigators, Satish Adwani

**Affiliations:** 1 Department of Genetics, Harvard Medical School, Boston, Massachusetts, USA; 2 Internal Medicine III, Cardiology, Department of Internal Medicine, Heidelberg University Hospital, Heidelberg, Germany; 3 Department of Pediatric Cardiology, Deutsches Herzzentrum der Charité, Berlin, Germany; 4 Experimental and Clinical Research Center, Corporate Member of Freie Universität Berlin and Humboldt-Universität zu Berlin, Charité - Universitätsmedizin Berlin, Berlin, Germany; 5 Institute of Cardiovascular Science, Centre for Paediatric Inherited and Rare Cardiovascular Disease, University College London, London, UK; 6 Centre for Inherited Cardiovascular Diseases, Great Ormond Street Hospital for Children NHS Trust, London, UK

**Keywords:** Genetic Testing, Genetics, Medical, Cardiomyopathies, Pediatrics, Genetic Diseases, Inborn

 The 2023 European Society of Cardiology (ESC) Guideline for the Management of Cardiomyopathies and the 2024 American Heart Association (AHA)/American College of Cardiology (ACC)/AMSSM/HRS/PACES/SCMR Guideline for the Management of Hypertrophic Cardiomyopathy (HCM)^1 2^ now recommend routine genetic testing for all children fulfilling diagnostic criteria for HCM. Guideline recommendations on phenotype description and the use of cardiac MR imaging have resulted in a change in clinical practice, but whether the same applies to genetic testing in childhood HCM is unknown.^3^ To understand current resource availability and clinical genetic testing practice, we performed a survey of centres caring for children with HCM within the International Paediatric Hypertrophic Cardiomyopathy Consortium (IPHCC), a large geographically diverse consortium of expert paediatric cardiomyopathy providers.^4^

An electronic survey was distributed to all members of the IPHCC (number of centres=42) between May 2024 and August 2024, of whom 34 centres from 14 countries (figure 1A) responded (online supplemental table 1). 20 centres (59%) were colocated with adult services and the number of HCM patients seen annually varied (<50 n=15 (44%), 50–100 n=10 (29%), >100 n=6 (18%), no information n=3 (9%)) (figure 1B). A third of centres reported an increase in the frequency of genetic testing (n=11, 32%) following changes in guideline recommendations^1 2^ (figure 1C). As a result, the majority (n=32, 94%) routinely offer genetic testing to all patients meeting diagnostic criteria for HCM (figure 1D). The provider of genetic testing varied across centres (geneticist n=16 (47%), cardiologist n=5 (15%), geneticist and cardiologist n=13 (38%) (figure 1E) and only two-thirds (n=20, 59%) routinely offer pretest genetic counselling (figure 1F). For most centres, genetic testing is funded by the health service or government (n=29, 78%), with a smaller number funded by health insurance (n=3, 8%) or the patient themselves (n=5, 15%). A similar number of centres perform testing exclusively for clinical purposes or in conjunction with research programmes (n=18 (53%) vs n=16 (47%)).

**Figure 1:**
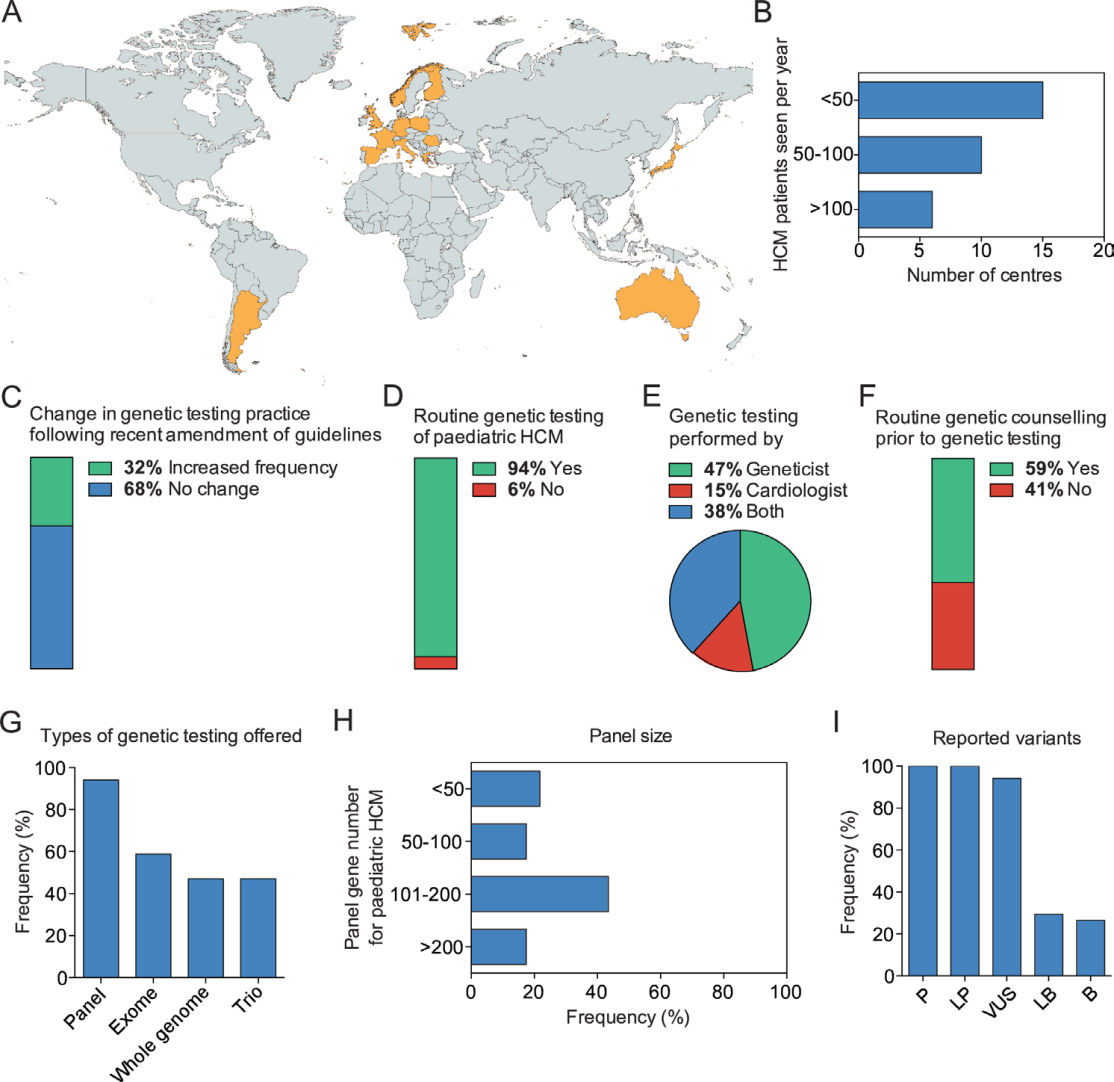
(A) Global map showing the distribution of responding centres (orange). Created with mapchart.net. The centres were located in Spain (n=6), UK (n=6), Italy (n=3), Romania (n=3), Australia (n=2), France (n=2), Germany (n=2), Japan (n=2), Poland (n=2), Argentina (n=1), Finland (n=1), Greece (n=1), Malta (n=1), Norway (n=1), unknown (n=1). (B) Bar graph showing the summary of the reported number of HCM patients seen per year for each centre. (C) Frequency chart showing the changes in the frequency of genetic testing in children with HCM after the recent guideline adjustments in the IPHCC centres. (D) Frequency chart of centres offering routine genetic testing to paediatric HCM patients. (E) Pie chart indicating the clinical specialty of the attending physician performing the genetic testing. (F) Frequency chart showing how many centres offer routine genetic counselling prior to genetic testing for paediatric HCM. (G) Frequency of genetic testing types offered by IPHCC centres for paediatric HCM. (H) Panel sizes used for panel sequencing in paediatric HCM. (I) Frequency of reported paediatric HCM variants by pathogenicity type across IPHCC centres. HCM, hypertrophic cardiomyopathy; IPHCC, International Paediatric Hypertrophic Cardiomyopathy Consortium.

The type of genetic testing performed by centres was varied, with many centres offering more than one type of testing. It included 32 (94%) offering panel sequencing, 20 (59%) exome sequencing, 16 (47%) whole genome sequencing and 16 (47%) trio-sequencing (figure 1G). When panel sequencing was used, panel size was variable between centres (<50 genes n=5 (16%), 50–100 genes n=4 (13%), 101–200 genes n=10 (31%), >200 genes n=4 (13%), unknown n=9 (28%)) (figure 1H). Two centres exclusively offer whole exome (n=1, 3%) or whole genome sequencing (n=1, 3%) to paediatric HCM patients. All centres routinely report pathogenic and likely pathogenic variants (n=34, 100%) and the majority report variants of uncertain significance (n=32, 94%). Likely benign and benign variants are reported by a smaller number of centres (n=10, 29% and n=9, 26%, respectively) (figure 1I).

This study describes the current use of genetic testing in a large geographically diverse consortium of expert paediatric cardiomyopathy providers. Important findings include a good availability of genetic testing for paediatric patients with HCM in this cohort but variability of genetic testing strategies and access to pretest genetic counselling. The survey did not evaluate access to post-test genetic counselling.

This survey demonstrated that a variety of strategies for genetic testing in childhood HCM are currently used, with significant variability in the size of gene panels. This may be related to institutional guidelines, laboratory affiliations and varying numbers of patients that present with non-isolated HCM seen in the centres surveyed. In addition, it is possible that centres where genetic testing is carried out in conjunction with research programmes may use broader testing platforms and/or larger panels for the discovery of rare genes.

The 2023 ESC Cardiomyopathy Guidelines^1^ and the 2024 AHA/ACC/AMSSM/HRS/PACES/SCMR HCM Guidelines^2^ differ in their definition of HCM, with the ESC adopting a broader, phenotype-driven definition that integrates syndromic, metabolic and neuromuscular aetiologies, which is especially relevant in paediatric cohorts. In contrast, the American guidelines focus more specifically on sarcomeric HCM and recommend restricting first-line genetic testing to sarcomeric genes. These varying approaches may partly explain the observed variation in panel size and testing strategy across centres in this survey.

Current genetic testing practices in paediatric HCM are largely in line with those in adult-onset HCM,^2 5^ although there was more frequent use of broader panels and exome/whole genome sequencing. This could reflect uncertainty about the best approach to genetic testing for children with cardiomyopathies. Previous studies have described the genetic architecture of idiopathic and familial paediatric cardiomyopathies, revealing a large overlap with adult-onset cardiomyopathies.^6 7^ However, most of these studies excluded children with identifiable syndromic, metabolic or neuromuscular causes, which account for a relevant minority of childhood cardiomyopathies, especially those with early disease manifestation and poor prognosis.^1 2^ Recent gene curation efforts by expert panels have identified genes with a strong evidence of causation for HCM, including syndromic disease,^8^ most of which would be included in standard cardiomyopathy gene panels.^9^ A relevant fraction of paediatric HCM is caused by mitochondriopathies. While some cardiomyopathy gene panels include nuclear DNA-encoded mitochondrial genes, they frequently omit genes from the mitochondrial genome (mtDNA). Moreover, even when whole exome or genome sequencing is used, pathogenic variants in mtDNA may go undetected. For HCM in adults, expanded testing using exome or whole genome sequencing has been shown to offer limited improvement in detection rates compared with targeted panel testing.^5^ As genetic causes of paediatric onset disease may be more diverse than adult-onset cardiomyopathies, expanded gene panels or even standardised first-line application of exome or whole genome sequencing in childhood-onset cardiomyopathy may be cost and outcome effective. In keeping with this, recent paediatric HCM studies incorporating exome or whole genome sequencing suggest potentially improved detection rates.^6 10^

Identification of a disease-causing variant (pathogenic or likely pathogenic) in an individual has important implications for management and family screening. However, in up to 40% of patients genetic testing may be inconclusive, creating ambiguity or uncertainty for families. This survey shows that in addition to disease-causing variants, many centres currently report variants of uncertain significance and almost a third report likely benign variants despite these being considered clinically inactionable. It is beyond the scope of this study to determine the best approach to reporting clinical genetic test results. One third of centres did not offer pretest genetic counselling, but the survey did not evaluate access to post-test genetic counselling, which can be invaluable in helping families interpret and understand genetic testing results. Availability of genetic professional support did not appear to be significantly associated with genetic testing strategy (online supplemental table 2–4).

In summary, this study reassuringly shows widespread access to genetic testing for children with HCM in a group of expert centres but with variability in clinical practices. These findings may not apply outside of expert centres, but they suggest that internationally-harmonised evidence-based recommendations for best practice of genetic testing in children with cardiomyopathies may be helpful to ensure resource-efficient and standardised clinical decision-making.

## Supplementary material

10.1136/jmg-2025-110696 online supplemental file 1
