# Supplementary material for: Contemporary practice and resource availability for genetic testing in paediatric hypertrophic cardiomyopathy
Source: J Med Genet. 2025 May 16;62(8):e110696. doi: 10.1136/jmg-2025-110696 (PMC12322417; doi:10.1136/jmg-2025-110696)
Supplement: online supplemental file 1 [file jmg-62-8-s001.pdf]

Supplementary File

Contemporary practice and resource availability for genetic testing in paediatric Hypertrophic Cardiomyopathy

Christoph Sandmann, MD, PhD[1,2,3*], Sabine Klaassen, MD[4,5,6,7,8], Juan Pablo Kaski, MD[9,10], Gabrielle Norrish, MD[9,10*]

On behalf of the International Paediatric Hypertrophic Cardiomyopathy Consortium

1 Department of Internal Medicine III (Cardiology, Angiology, and Pneumology), Heidelberg, Germany

2 German Center for Cardiovascular Research, Partner Site Heidelberg/Mannheim, Heidelberg, Germany

3 Department of Genetics, Harvard Medical School, Boston, MA, USA

4 Charité – Universitätsmedizin Berlin, corporate member of Freie Universität Berlin and Humboldt-Universität zu Berlin, Berlin, Germany

5 Experimental and Clinical Research Center, Lindenberger Weg 80, Berlin, Germany

6 Max Delbrück Center for Molecular Medicine in the Helmholtz Association (MDC), Berlin, Germany

7 DZHK (German Centre for Cardiovascular Research), partner site Berlin, Berlin, Germany

8 Department of Pediatric Cardiology, Deutsches Herzzentrum der Charité (DHZC), Berlin, Germany

9 Centre for Paediatric Inherited & Rare Cardiovascular Disease, Institute of Cardiovascular Science, London, United Kingdom

10 Centre for Inherited Cardiovascular Diseases, Great Ormond Street Hospital, London, United Kingdom

Supplementary Table 1. Survey sent to the participating centres.

1. What country is your centre in?
2. How many patients with HCM does your centre see each year?
3. Is your centre co-located with adult services?
4. Does your centre routinely offer genetic testing to patients meeting diagnostic criteria for HCM?
5. Did the recent changes of ESC recommendations for genetic testing in children change your practice in genetic testing of paediatric cases?
6. On what basis is genetic testing typically performed in your centre?
7. How is genetic testing funded at your centre?
8. What type of genetic testing is typically performed/available at your centre?
9. If panel testing is used, how many genes are included?
10. What class of variant are routinely reported?
11. Who performs genetic testing at your centre?
12. Do your patients routinely see a genetics counsellor or geneticist prior to genetic testing at your centre?

Supplementary Table 2. Association between the genetic testing platform used and the speciality of the testing physicians.

	**only panel sequencing used**	**additional genetic testing platforms used**
**Geneticist**	4	12
**Cardiologist**	2	3
**Both**	4	9

Fisher´s exact test: $p = 0.8809$

Supplementary Table 3. Association between the reporting of VUS and genetic professional support.

	**Reporting of VUS**	**No reporting of VUS**
**Genetic professional support (geneticist involved in testing and/or avialable pre-test genetic counceling)**	27	2
**No genetic professional support**	5	0

Fisher´s exact test: $p < 0.999$

Supplementary Table 4. Association between the reporting of LB/B variants and genetic professional support.

	**Reporting of LB/B variants**	**No reporting of LB/B variants**
**Genetic professional support (geneticist involved in testing and/or avialable pre-test genetic counceling)**	9	20
**No genetic professional support**	1	4

Fisher´s exact test: $p < 0.999$
